# Identification of diagnostic model in heart failure with myocardial fibrosis and conduction block by integrated gene co-expression network analysis

**DOI:** 10.1186/s12920-024-01814-w

**Published:** 2024-02-14

**Authors:** Yonghua Yuan, Yiwei Niu, Jiajun Ye, Yuejuan Xu, Xuehua He, Sun Chen

**Affiliations:** 1 Department of Pediatrics, Institute of Pediatric Research, Children’s Hospital of Soochow University, Suzhou, Jiangsu China; 2 Department of Pediatric Cardiology, Hunan Provincial People’s Hospital, The First Affiliated Hospital of Hunan Normal University, Changsha, China; 3 Department of Pediatric Cardiology, Xinhua hospital, School of medicine, Shanghai Jiaotong university, Shanghai, China

**Keywords:** Heart failure, Biomarkers, Bioinformatics, Myocardial fibrosis, Conduction block, Gene expression synthesis

## Abstract

**Background:**

Despite the advancements in heart failure(HF) research, the early diagnosis of HF continues to be a challenging issue in clinical practice. This study aims to investigate the genes related to myocardial fibrosis and conduction block, with the goal of developing a diagnostic model for early treatment of HF in patients.

**Method:**

The gene expression profiles of GSE57345, GSE16499, and GSE9128 were obtained from the Gene Expression Omnibus (GEO) database. After merging the expression profile data and adjusting for batch effects, differentially expressed genes (DEGs) associated with conduction block and myocardial fibrosis were identified. Gene Ontology (GO) resources, Kyoto Encyclopedia of Genes and Genomes (KEGG) resources, and gene set enrichment analysis (GSEA) were utilized for functional enrichment analysis. A protein-protein interaction network (PPI) was constructed using a string database. Potential key genes were selected based on the bioinformatics information mentioned above. SVM and LASSO were employed to identify hub genes and construct the module associated with HF. The mRNA levels of TAC mice and external datasets (GSE141910 and GSE59867) are utilized for validating the diagnostic model. Additionally, the study explores the relationship between the diagnostic model and immune cell infiltration.

**Results:**

A total of 395 genes exhibiting differential expression were identified. Functional enrichment analysis revealed that these specific genes primarily participate in biological processes and pathways associated with the constituents of the extracellular matrix (ECM), immune system processes, and inflammatory responses. We identified a diagnostic model consisting of 16 hub genes, and its predictive performance was validated using external data sets and a transverse aortic coarctation (TAC) mouse model. In addition, we observed significant differences in mRNA expression of 7 genes in the TAC mouse model. Interestingly, our study also unveiled a correlation between these model genes and immune cell infiltration.

**Conclusions:**

We identified sixteen key genes associated with myocardial fibrosis and conduction block, as well as diagnostic models for heart failure. Our findings have significant implications for the intensive management of individuals with potential genetic variants associated with heart failure, especially in the context of advancing cell-targeted therapy for myocardial fibrosis.

**Supplementary Information:**

The online version contains supplementary material available at 10.1186/s12920-024-01814-w.

## Background

Heart failure (HF) manifests as a clinical syndrome where individuals experience difficulty breathing or have limitations in physical activity because of the inadequate filling or expulsion of blood from the ventricles [1]. The worldwide incidence of HF has increased considerably, affecting approximately 23 million people presently, and this figure is estimated to surpass 30 million by 2030 [2]. In the population aged 55 and above, the post-diagnosis survival rate over a five-year period ranges from 20 to 50%, establishing HF as the leading cause of death among older adults [3–5].

Several studies [6–8] have demonstrated that individuals with HF display varying degrees of myocardial fibrosis, and the severity of fibrosis is directly correlated with prognosis. In addition to causing mechanical impairments, myocardial fibrosis also contributes to disruptions in electrical conduction throughout the myocardium [9]. Irregularities in cardiac electrical activity are frequently observed in HF patients. About one-third of individuals affected by HF exhibit ventricular conduction abnormalities, excluding atrioventricular block and intra-atrial block [1, 10]. Consequently, the prevention and inhibition of myocardial fibrosis and conduction block have become prominent topics in HF research.

In the last 40 years, significant progress has been achieved in the management of HF through the use of drugs that decrease long-term mortality and rehospitalization rates in HF patients by inhibiting the development of myocardial fibrosis [8, 11, 12]. Recent studies employing modified T cells targeting cardiac fibrosis have shown promising outcomes [13]. However, an essential challenge in the clinical application of engineered T cell intervention is the identification of individuals who require it. Traditional diagnostic techniques like echocardiography, cardiac MRI, and BNP/NT-proBNP levels have limited efficacy in early detection of pathophysiological changes associated with HF. Hence, the development of reliable diagnostic models for HF is imperative for its prevention and treatment.

With the increasing abundance of gene expression profile data, the opportunity to construct a diagnostic model for HF is becoming more convenient. In a study by Tian, Y et al. [14], a diagnostic model for HF was developed using a combination of random forest and artificial neural network. From this study, *HMOX2*, *SERPINA3*, *LCN6*, *CSDC2*, *FREM1*, and *ZMAT1* were identified as the hub genes of the model. Another research project carried out an analysis of weighted gene co-expression networks to establish a model for identifying HF, pinpointing *CUX1* and *ASB1* as the hub genes contributing to the disease [15]. Furthermore, Niu, X et al. [16] also utilized weighted gene co-expression network analysis to identify a diagnostic model for HF after acute myocardial infarction, which included six crucial genes involved in the processes of inflammation, immunity, and apoptosis. These studies primarily focused on the discovery of HF biomarkers, overlooking the two fundamental phenotypes of HF - myocardial fibrosis and conduction block.

The primary objective of this study is to create a diagnostic framework for identifying HF by investigating the distinctive genes linked to myocardial fibrosis and conduction block. To attain this objective, we conducted a thorough examination of three datasets (GSE16499, GSE57345, and GSE9128) and obtained pertinent differential genes. Analysis of enrichment unveiled that these differential genes are primarily associated with the activation of immune cells and the initiation of inflammatory responses. Subsequently, we devised a diagnostic model for HF, which revealed that the hub genes are closely linked to immune infiltration. This discovery bears significant clinical implications for the early detection, prevention, and treatment of HF.

**Fig. 1:**
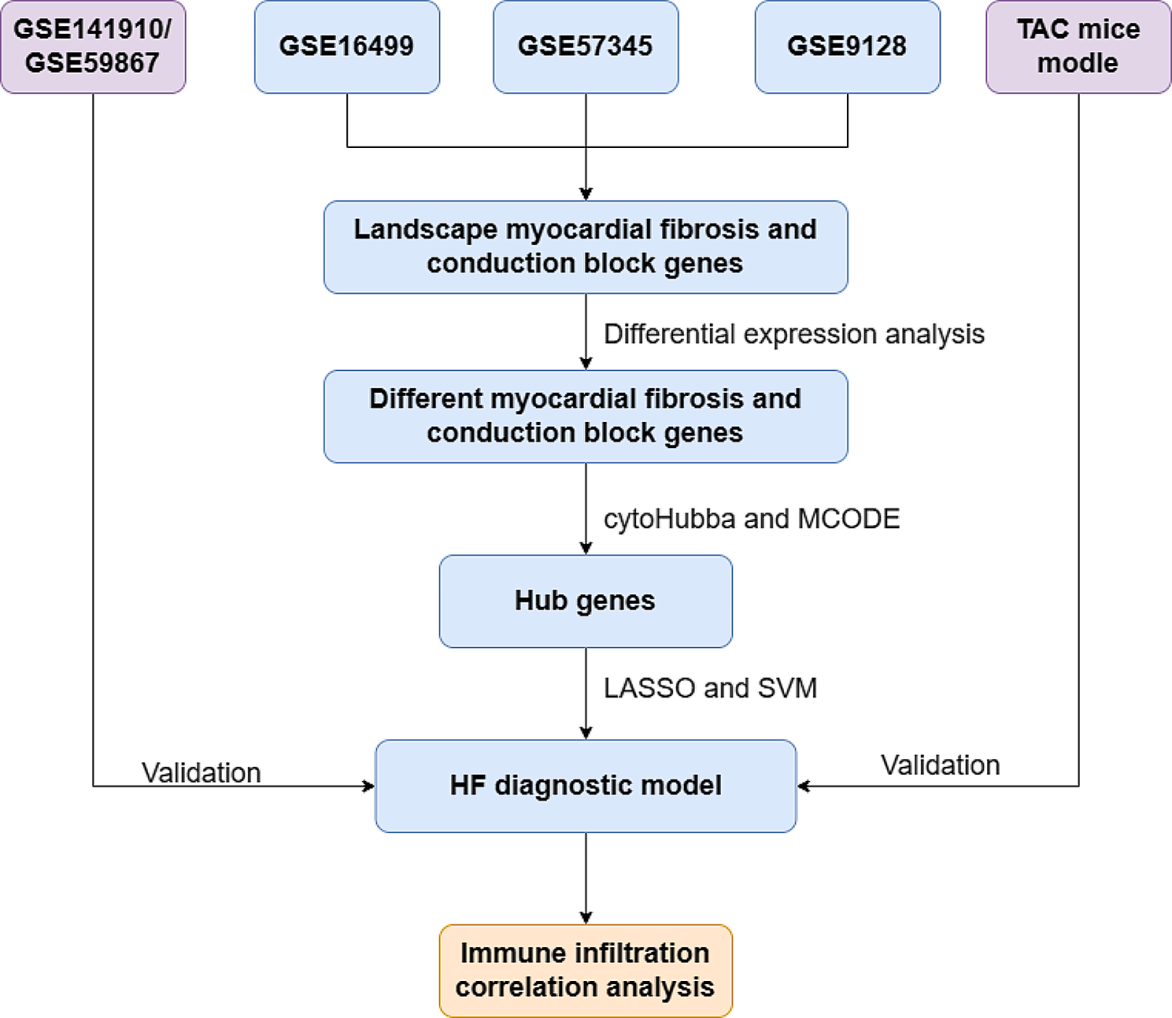
Study flowchart TAC, transverse-aortic constriction; LASSO, least absolute shrinkage and selection operator; SVM, support vector machines; HF, heart failure

## Materials and methods

### Data collection and processing

The entire process flow is presented in Fig. 1. The gene expression profiles of GSE57345, GSE16499, and GSE9128 were obtained from the GEO database available at [http://www.ncbi.nlm.nih.gov/geo/]. GSE57345 and GSE16499 consisted of samples obtained from cardiac tissues, while GSE9128 consisted of a sample from peripheral blood mononuclear cells (refer to Table 1). Within GSE16499, there were 15 non-failure controls (NF) and 15 HF patient samples resulting from ischemic cardiomyopathy (ICM). In GSE57345, there were 136 NF samples and 177 HF patient samples, including 82 samples resulting from Non-ischemic cardiomyopathy (NICM) and 95 caused by ICM. As for GSE9128, there were 3 NF samples and 8 HF patient samples, with 4 samples caused by NICM and 4 caused by ICM. After merging and correcting for batch effects in the three datasets mentioned earlier, the ComBat function from the SVR package [17] was employed to remove any remaining batch effects.

In order to identify genes associated with Conduction block and Myocardial Fibrosis, we performed a search on GeneCards using the keywords ‘Conduction block’ and ‘Myocardial Fibrosis’. Subsequently, we compared the genes related to Conduction block and myocardial fibrosis that we obtained from GeneCards with the genes present in the three aforementioned datasets. By intersecting these two sets of genes, we obtained the final list of genes that are associated with both Conduction block and myocardial fibrosis.

**Table 1:** Datasets and sample information from GEO

GSE series	Platform	Gene Count	Heart Failure (*n* = 790)		Normal (*n* = 366)	Total (*n* = 1156)	Samples Type
			NICM	ICM			
**Train Set**							
GSE16499	GPL5175	15,876	0	15(1.90%)	15(4.10%)	30(2.59%)	Heart tissue
GSE57345	GPL11532	20,254	82(10.38%)	95(12.03%)	136(37.16%)	313(27.08%)	Heart tissue
GSE9128	GPL96	13,096	4(0.505%)	4(0.505%)	3(0.82%)	11(0.95%)	peripheral blood
**Validation Set**							
GSE59867	GPL6244	23,307	0	390(49.37%)	46(12.57%)	436(37.72%)	peripheral blood
GSE141910	GPL16791	17,102	200(25.32%)	0	166(45.35%)	366(31.66%)	Heart tissue

### Identification of DEGs and their functional enrichment analysis

The R package ‘limma’ was used to identify differentially expressed genes (DEGs) between NF and HF patient samples. Upregulated DEGs were defined as having a log2 fold change (FC) value greater than 1.2 and an adjusted P value less than 0.05, while downregulated DEGs were defined as having a log2 FC value less than 0.83 and an adjusted P value less than 0.05. The DEGs were then subjected to GO, KEGG enrichment analysis [18], and GSEA using the R package ‘clusterProfiler’. The GO function was evaluated in terms of biological process (BP), molecular function (MF), and cellular component (CC). A statistically significant adjusted P-value less than 0.05 was considered.

### Protein interaction network (PPI) and module screening

To construct a protein-protein interaction (PPI) network of the differentially expressed genes (DEGs), we utilized the STRING database (http://stringdb.org/). The network was built with a combined score of at least 900 [19]. The Network Analyzer tool in Cytoscape [20] was employed to calculate the attributes of the network nodes. Hub nodes in the network were identified based on a connectivity degree of at least 10, considering the degree of the node, tightness, and maximum cluster center. Subsequently, the MCODE plugin was used to identify important clusters and genes, with a degree cutoff of 2, K-cor of 2, and node score cutoff of 0.2.

### Construction and verification of HF diagnostic model

The samples were randomly divided into a training set and an internal validation set with a ratio of 7:3. The diagnostic model was constructed using the least absolute shrinkage and selection operator (LASSO) regression analysis and support vector machine (SVM) method. The ‘glmnet’ [21] and e1071 packages in the R package [22] were used for the LASSO regression analysis and SVM method, respectively, in the training set. The area under the ROC curves (AUC) was calculated and calibration curves were plotted in both the training set and internal validation set. Gene signatures with an AUC value > 0.5 in the internal validation set were retained. The beta coefficients and truncation value from the LASSO model were extracted to construct the HF diagnostic model. The performance of the diagnostic model was evaluated by calculating the AUC of the ROC in both the internal and external validation datasets (GSE21125 and GSE59867).

### Immune infiltration analysis

Using the leukocyte gene signature set (LM22) provided by CIBERSORT [23], we determined the composition ratio of 22 immune cells in each sample. We then investigated the correlation between the diagnostic model genes and the 22 types of immune cells using the IOBR function in the R package [24].

### Real time quantitative PCR

Eight-week-old male C57BL/6 mice were randomly divided into two groups: sham (*n* = 6) and TAC (*n* = 6) and obtained from Xinhua Hospital, Shanghai Jiao Tong University. TAC model mice were established based on a previous study [25]. After four weeks of TAC, mice were sacrificed by administration of intraperitoneal sodium pentobarbital, and heart tissue specimens were harvested. Total RNA was extracted from fresh-frozen tissues using TRIZOL reagent (Takara, Kyoto, Japan). The frozen heart tissues were also used for total RNA extraction using Trizol (Takara, Kyoto, Japan), and cDNA was reversely transcribed using a PrimeScript™ RT Reagent Kit (Takara) following the manufacturer’s instructions. The primer sequences are listed in Table 2.

**Table 2:** Primers sequence

Genes	Forward	Reverse
IFIT2	AGTACAACGAGTAAGGAGTCACT	AGGCCAGTATGTTGCACATGG
IFITM2	TGGGCTTCGTTGCCTATGC	AGAATGGGGTGTTCTTTGTGC
OAS2	TTGAAGAGGAATACATGCGGAAG	GGGTCTGCATTACTGGCACTT
EGR1	TCGGCTCCTTTCCTCACTCA	CTCATAGGGTTGTTCGCTCGG
STAT3	CAATACCATTGACCTGCCGAT	GAGCGACTCAAACTGCCCT
JAK1	CTCTCTGTCACAACCTCTTCGC	TTGGTAAAGTAGAACCTCATGCG
JAK2	TTGTGGTATTACGCCTGTGTATC	ATGCCTGGTTGACTCGTCTAT
CXCL12	TGCATCAGTGACGGTAAACCA	TTCTTCAGCCGTGCAACAATC
PDGFRB	TTCCAGGAGTGATACCAGCTT	AGGGGGCGTGATGACTAGG
FGR	CGGCTGAAGAACGCTATTTCC	GGGCGACGAATATGGTCACTC
IL10	GCTCTTACTGACTGGCATGAG	CGCAGCTCTAGGAGCATGTG
TLR4	ATGGCATGGCTTACACCACC	GAGGCCAATTTTGTCTCCACA
HIF1A	ACCTTCATCGGAAACTCCAAAG	ACTGTTAGGCTCAGGTGAACT
ITGA3	CCTCTTCGGCTACTCGGTC	CCGGTTGGTATAGTCATCACCC
ITGAL	CCAGACTTTTGCTACTGGGAC	GCTTGTTCGGCAGTGATAGAG
ITGA8	TGGCTGGGATTCCAAGAGGA	GTGCCCCGACCAATATGTCA

### Statistical analysis

The statistical calculations were performed using R language (version 4.0.2). An independent t-test was used to compare continuous variables between two groups. For independent variables with non-normal distribution, the Mann-Whitney U test (Wilcoxon rank-sum test) was conducted. The ROC package pROC was utilized to plot ROC curves and calculate the area under the curve (AUC) for assessing the performance of the HF diagnostic model. All statistical P values were bilateral, and a significance level of *P* < 0.05 was considered statistically significant.

## Results

### Identification of conduction block and myocardial fibrosis-related DEGs

**Fig. 2:**
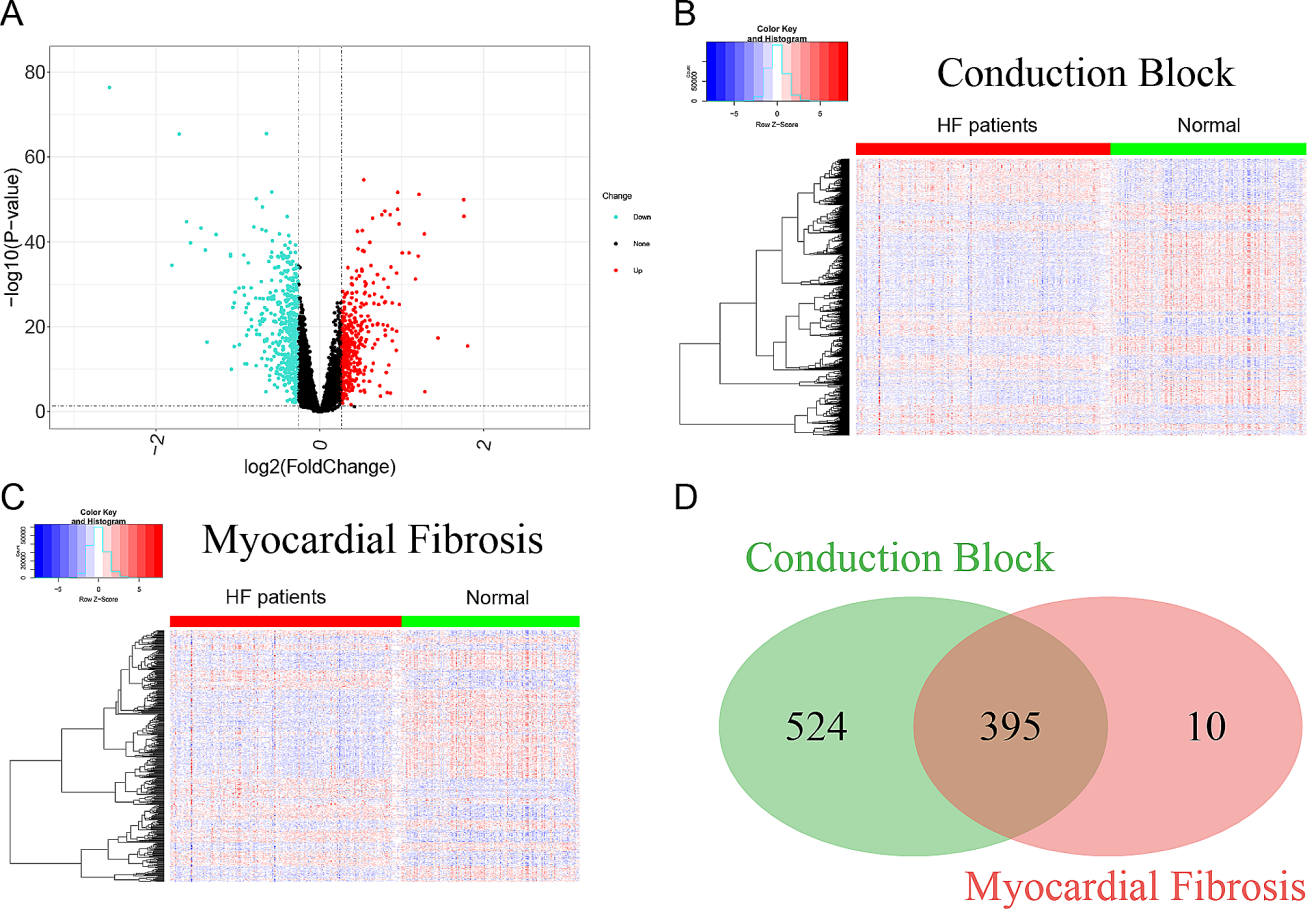
Identification of DEGs with conduction block and myocardial fibrosis. (**A**) Volcano plot of significant DEGs with conduction block or myocardial fibrosis. (**B**) Heatmap of DEGs with conduction block. (**C**) Heatmap of DEGs with myocardial fibrosis. (**D**) Venn diagram of DEGs with conduction block and myocardial fibrosis

In the study, three datasets were used to identify differentially expressed genes (DEGs). By applying ‘Batch correction’, a total of 929 DEGs were identified, with 400 genes upregulated and 529 genes downregulated (Data preprocessing see Figure S1). Further analysis revealed that 395 DEGs were related to conduction block and myocardial fibrosis. These DEGs were visualized using a volcano map (Fig. 2A), heatmap (Fig. 2B, C), and Venn diagram (Fig. 2D).

### The DEGs are enriched in the process of ECM, inflammatory response and immune cell activation

**Fig. 3:**
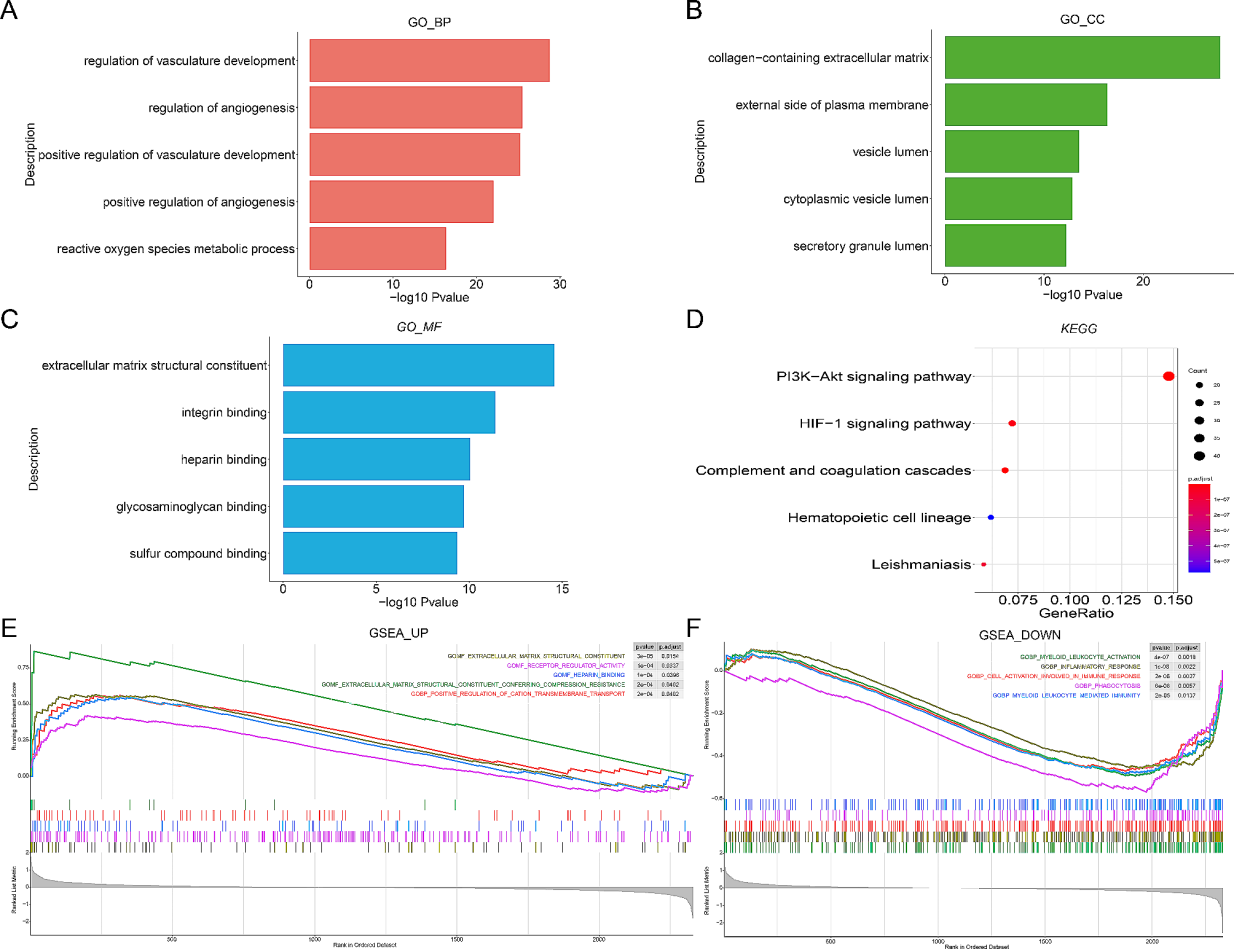
Biological differences between normal and patient samples. (**A**) Bar plot of top 5 enriched GO terms of DEGs in BP. (**B**) Bar plot of top 5 enriched GO terms of DEGs in CC. (**C**) Bar plot of top 5 enriched GO terms of DEGs in MF. (**D**) Bubble plot of significantly enriched KEGG pathways of DEGs. (**E**) Top 5 enriched GSEA pathway of DEGs for up-regulation part. (**F**) Top 5 enriched GSEA pathway of DEGs for down-regulation part

To investigate the biological processes and functions of DEGs in conduction block and myocardial fibrosis, we performed Gene Ontology (GO), Kyoto Encyclopedia of Genes and Genomes (KEGG), and Gene set enrichment analysis (GSEA) on these DEGs (Fig. 3, A-F). The GO annotations of DEGs included BP (biological process), CC (cellular component), and MF (Molecular function), which were utilized to analyze the functional enrichment of DEGs. The DEGs were primarily associated with angiogenesis and substance transport, such as regulation of vasculature development, extracellular matrix structural constituent, and integrin binding (Fig. 3, A-C). KEGG analysis was performed to determine the relationship between DEGs and signaling pathways. The DEGs were found to be mainly involved in cell proliferation and immune processes, including the PI3K-Akt signaling pathway, HIF-1 signaling pathway, Leishmaniasis, and Hematopoietic cell lineage (Fig. 3D). GSEA supported the findings of the GO and KEGG analysis (Fig. 3E, F). Overall, the DEGs exhibited significant associations with the extracellular matrix (ECM), inflammatory response, and immune cell activation.

### Sixteen hub genes were selected to construct a diagnostic model for HF

**Fig. 4:**
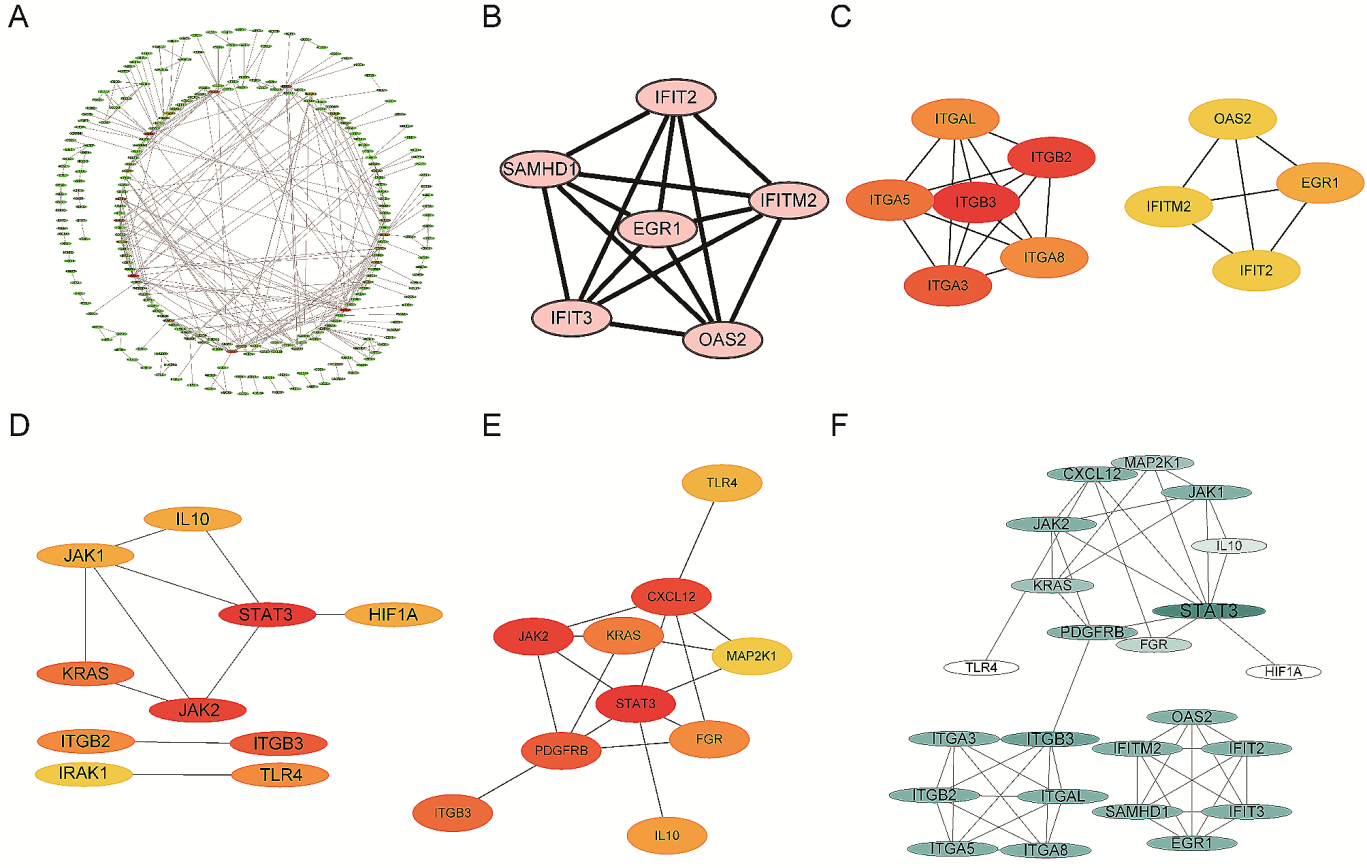
PPI network and hub gene screening. (**A**) PPI network of 395 DEGs with conduction block and myocardial fibrosis phenotypes. (**B**) Top scoring module genes screened by MCODE. (**C**) Top 10 genes screened by MCC algorithm in cytoHubba. (**D**) Top 10 genes screened by Degree in cytoHubba. (**E**) Top 10 genes screened by Closeness in cytoHubba. (**F**) PPI network of 23 hub genes. The node color reflects the degree; the greater the degree, the darker the node color

To analyze the interactions of differentially expressed genes (DEGs), protein-protein interaction (PPI) networks were constructed using the STRING database and visualized using Cytoscape software (Fig. 4.A). Important node genes and subnetworks were further analyzed using the Cytohubba and MCODE plugins, respectively. By overlapping the four modules, a total of twenty-three candidate hub genes were identified (Fig. 4.B-F).

**Fig. 5:**
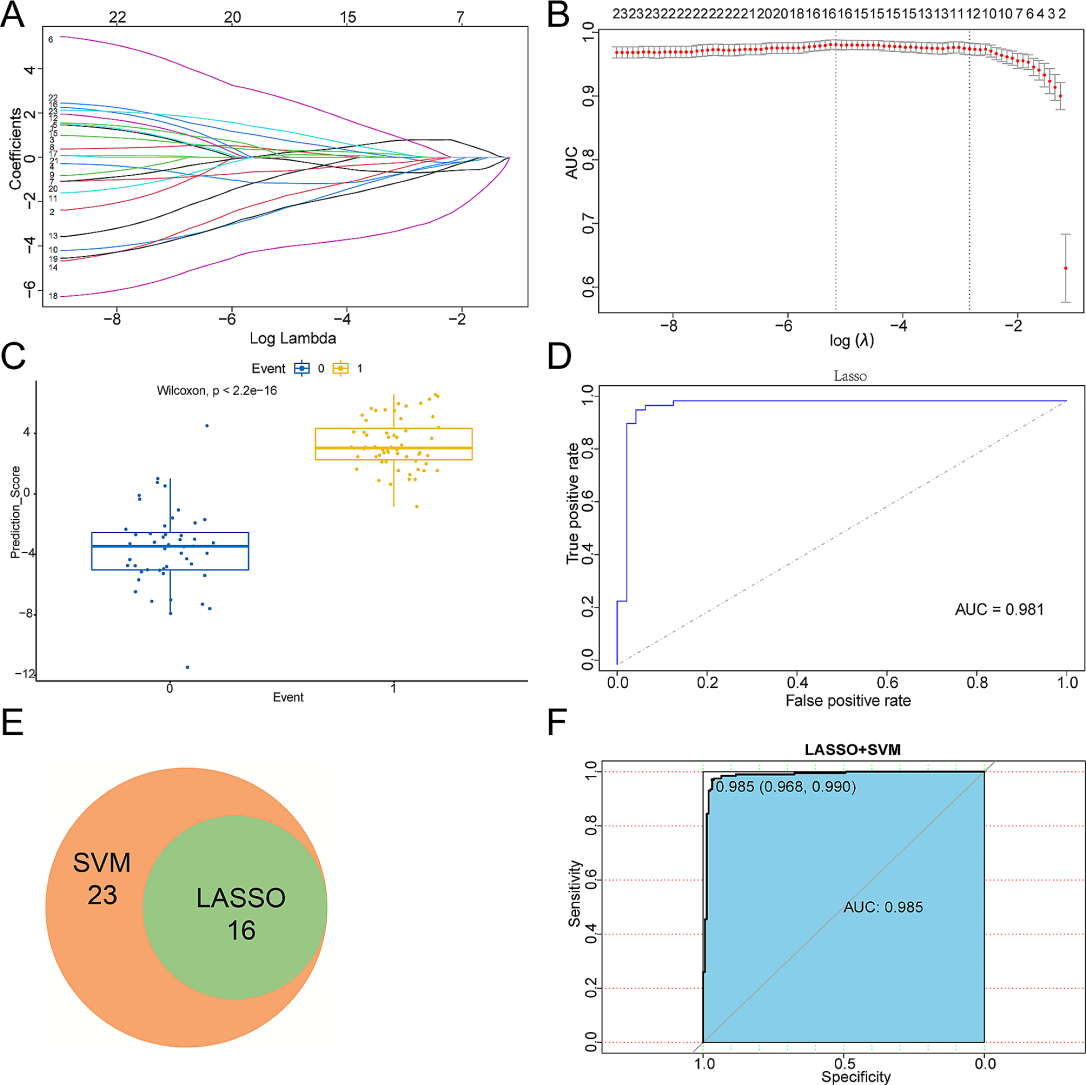
Construction of HF diagnostic model by LASSO and SVM. (**A**) LASSO coefficient profiles of candidate genes. (**B**) The optimal tuning parameter log (Lambda) in LASSO regression analysis. (**C**) Comparison of LASSO prediction score between normal and HF patient samples. (**D**) ROC curve evaluation of LASSO regression analysis. (**E**) Venn diagram demonstrating overlapping key feature genes screened by LASSO and SVM. (**F**) ROC curve evaluation of LASSO + SVM

Using LASSO, we identified 16 out of the 23 candidate signature genes, with a lambda.min value of 0.005714653 (Fig. 5, A-B). In the internal validation sets, these 16 candidate signature genes effectively distinguished between HF patient samples and control samples, achieving an AUC of 0.981 (Fig. 5, C-D). The support vector machine (SVM) analysis retained all 23 hub genes with an AUC greater than 0.5 (Fig. 5E).

By overlapping the genes from LASSO and SVM, we identified a robust signature gene set consisting of 16 genes (Fig. 5E; Table 3). As indicated in Table 3, the genes *IFIT2, IFITM2, OAS2, STAT3, JAK1, JAK2*, and *IL10* play a significant role in immune response and inflammatory processes. On the other hand, the genes *FGR, TLR4, HIF1A, CXCL12, ITGA3, ITGAL, ITGA8, PDGFRB*, and *EGR1* primarily participate in cell adhesion, migration, and chemotaxis. The coefficients and truncation values from Lasso analysis were used to construct the diagnostic model (Table 3). The diagnostic model in the training set demonstrated excellent performance with an AUC value of 0.985 (Fig. 5F).

**Table 3:** Parameters and functional clustering of diagnostic model gene

functional clustering	Model gene	β value	p value
Immune response/ inflammatory response	IL10	-1.812	3.21E-21
	TLR4	+ 0.111	5.54E-08
	IFIT2	+ 0.180	3.87E-35
	IFITM2	-1.165	6.80E-25
	OAS2	+ 2.713	2.65E-16
	STAT3	− 0.076	2.95E-38
	JAK1	-4.070	5.31E-40
	JAK2	+ 0.324	3.99E-22
Cell adhesion/ migration/ chemotaxis	FGR	-0.822	1.13E-12
	HIF1A	-2.320	3.45E-24
	CXCL12	+ 0.096	1.26E-19
	ITGA3	-0.624	8.34E-20
	ITGAL	+ 0.810	1.85E-11
	ITGA8	+ 1.217	4.63E-08
	PDGFRB	-2.372	2.91E-20
	EGR1	+ 0.332	7.84E-08
Constant value	72.002		

### The HF diagnostic model was validated by external dataset and the hub genes upregulated in TAC mouse model.

**Fig. 6:**
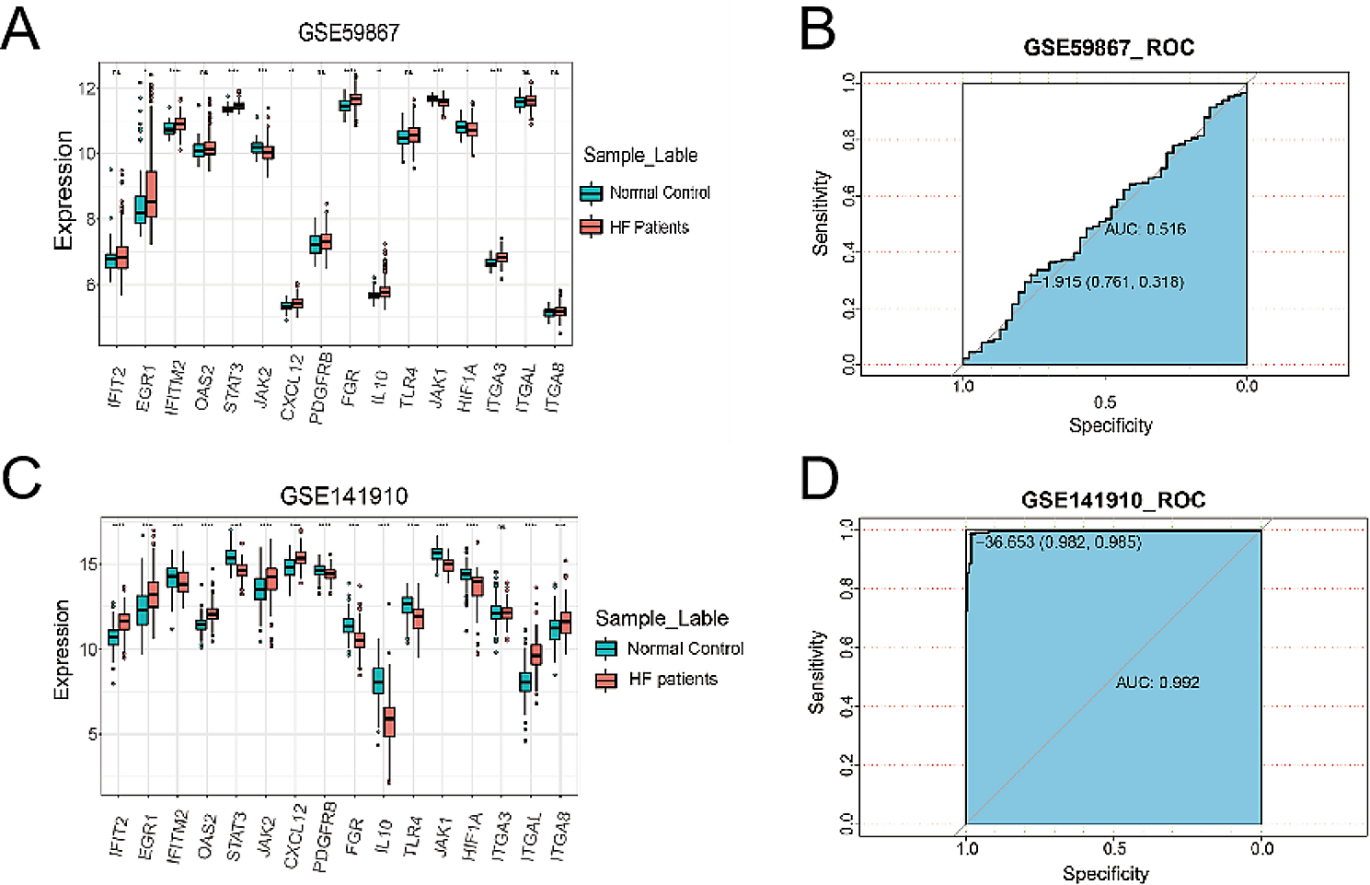
Validation of diagnostic model in external datasets. (**A**) Boxplots of the expression levels of diagnostic model gene in normal control and HF patient samples from GSE59867. (**B**) ROC curve for diagnostic model performance in GSE59867. (**C**) Boxplots of the expression levels of diagnostic model gene in normal and HF patient samples from GSE141910. (**D**) ROC curve evaluation of diagnostic model performance in GSE141910. ns *p* > 0.05, *p < 0.05, **p < 0.01, ***p < 0.001, ****p < 0.0001

To evaluate the accuracy and applicability of the diagnostic model, we conducted validation using two external datasets: GSE141910 and GSE59867. GSE141910 consisted of cardiac tissue samples from NICM patients, while GSE59867 included peripheral blood mononuclear cell samples from ICM patients. Remarkably, out of the 16 central genes, 10 exhibited distinct expression patterns in the HF and control samples in both externally validated datasets (see Fig. 6A, C). The AUC value of GSE141910 was 0.992 (Fig. 6.D), indicating strong performance of the diagnostic model. However, the AUC value of GSE59867 was only 0.516 (Fig. 6.B), possibly due to variations in expression patterns between heart tissue and peripheral blood mononuclear cells.

**Fig. 7:**
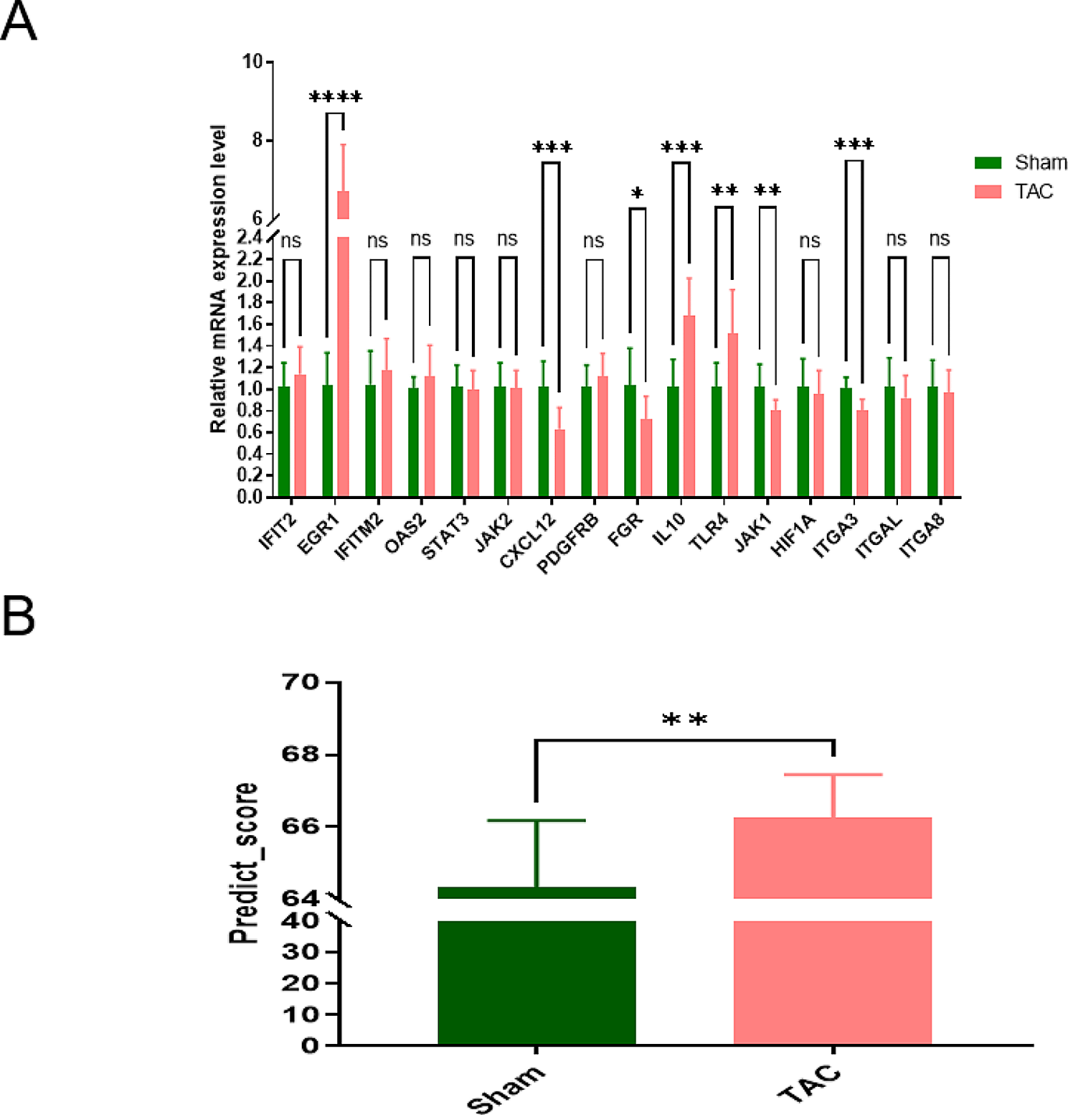
Validation of diagnostic model in TAC mouse model. (**A**) Real-time PCR analyses of the expression levels of diagnostic model genes in heart tissue from TAC and sham group mice. (**B**) Comparison of prediction score of diagnostic model between TAC and sham group mice. PCR, polymerase chain reaction; TAC, transverse-aortic constriction. ns *p* > 0.05, *p < = 0.05, **p < = 0.01, ***p < = 0.001, ****p < = 0.0001

To further validate the diagnostic model, we performed tests on TAC mice and analyzed the mRNA expression levels of the 16 genes in cardiac tissues. Out of these genes, 7 showed distinct expression patterns between the TAC group and the sham group (*p* < 0.05) (Fig. 7A). Moreover, the prediction score of the diagnostic model also displayed a significant difference between the TAC group and the sham group, indicating that the hub genes could potentially serve as indicators of cardiac dysfunction in mice as well (Fig. 7B).

### The relationship between diagnostic model and immune cells infiltration

**Fig. 8:**
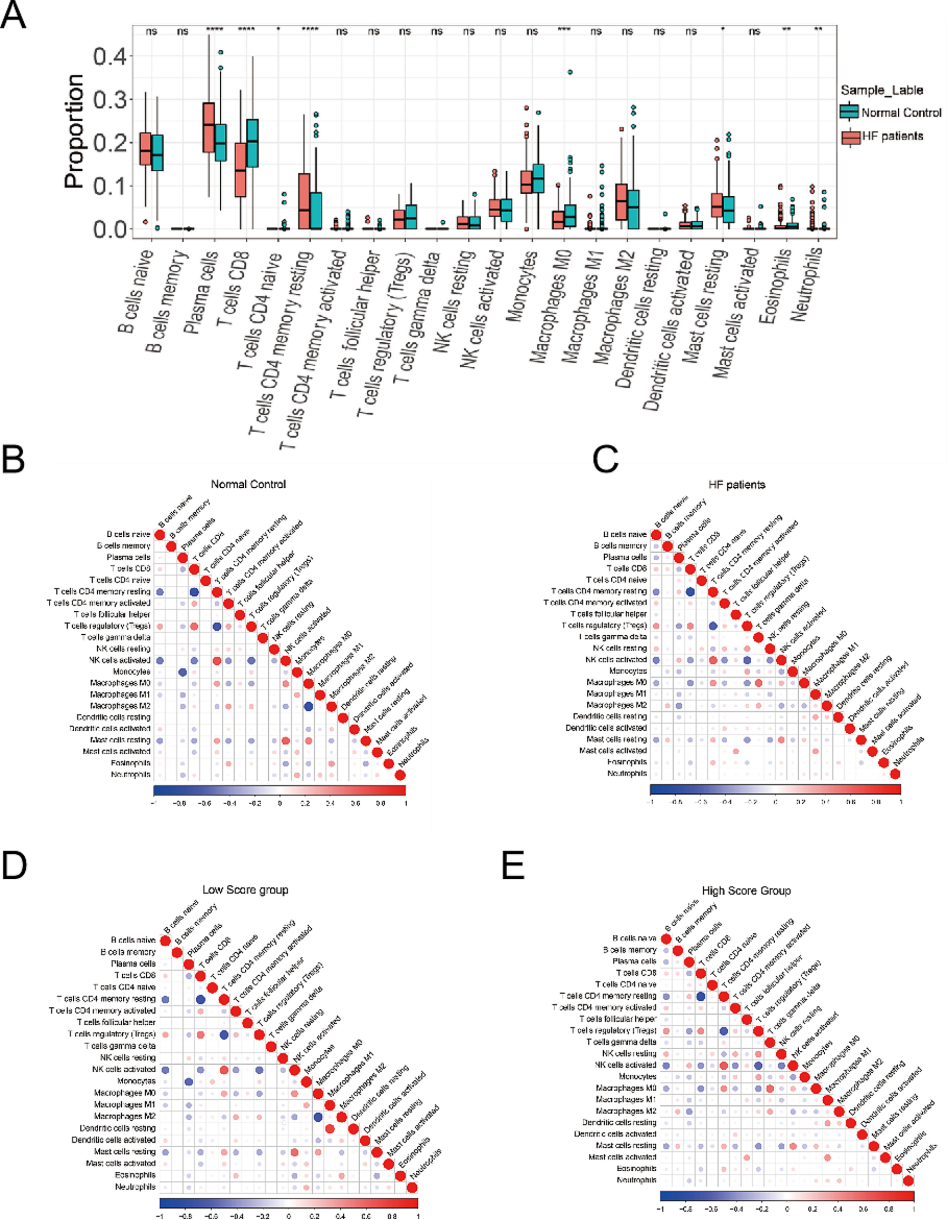
The relationship between diagnostic model and immune cells infiltration. (**A**) Stacked histogram of 22 types of immune cell infiltration corresponding to the dataset(**B**) Boxplot of infiltration of 22 immune cells in normal control samples. (**C**) Correlation heatmap of immune cells in HF patient samples. (**D**) Correlation heatmap of immune cells in low score group of diagnostic model. (**E**) Correlation heatmap of immune cells in high score group of diagnostic model. HF, heart failure; ns *p* > 0.05, *p < = 0.05, **p < = 0.01, ***p < = 0.001,****p < = 0.0001

Table 3 presents the results of the analysis of 16 hub genes, showing that 11 of them are associated with inflammatory responses and immune activation processes. In order to compare the immune reactions between HF patients and NF patients, an analysis of immune cell infiltration was conducted. The analysis revealed significant differences in the infiltration of 22 types of immune cells between the two patient groups (Fig. 8.A-B), particularly in plasma cells, CD8 + T cells, CD4 + T cells, and resting memory CD4 + T cells. It is worth noting that there were variations in the level of immune infiltration observed between the groups. For example, in normal samples, there was a significant negative correlation between macrophages M2 and resting dendritic cells, whereas a weak positive correlation was observed in the HF group (Fig. 8.C-D). Furthermore, the differences in immune cell infiltration observed in the high and low score subgroups, based on the predicted score of the diagnostic model, were similar to those observed in the two groups mentioned above (Fig. 8.E-F). This side-by-side comparison demonstrates the accuracy of the diagnostic model.

**Fig. 9:**
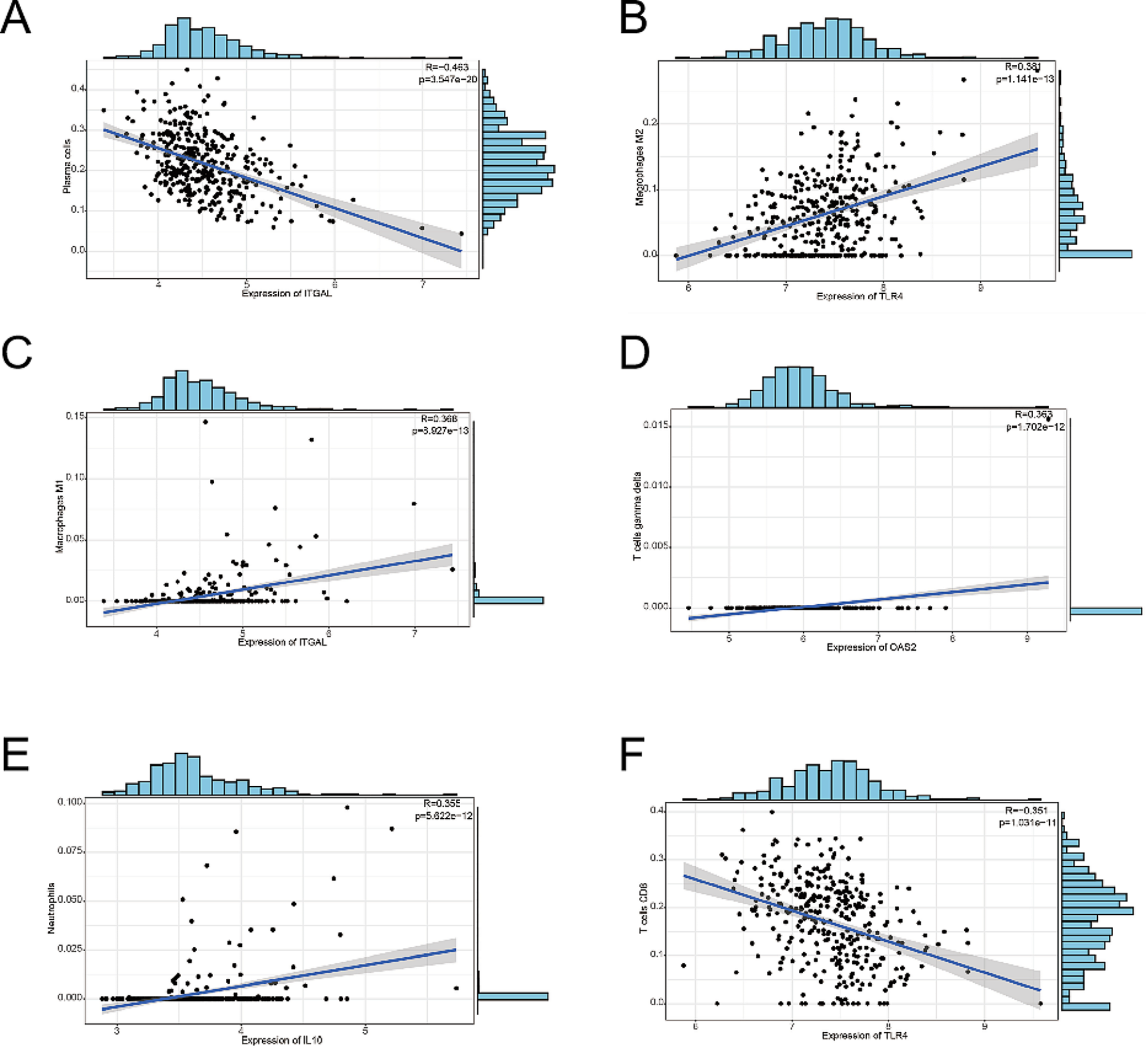
Correlation between diagnostic model genes and immune cells. (**A**)Scatter plot showed the association between ITGAL and Plasma cells. (**B**) Scatter plot showed the association between TLR4 and Macrophages M2. (**C**) Scatter plot showed the association between ITGAL and Macrophages M1. (**D**) Scatter plot showed the association between OAS2 and T cell gamma/delta. (**E**) Scatter plot showed the association between IL10 and Neutrophils. (**F**) Scatter plot showed the association between TLR4 and T cells CD8

The correlations revealed that TLR4 and ITGAL were associated with different immune cells (Fig. 9.A-F). Notably, TLR4 exhibited a negative correlation with CD8 + T cells and a positive correlation with MacrophagesM2. These findings indicate that the infiltration of immune cells may have a significant impact on the progression of myocardial fibrosis and conduction block in HF.

## Discussion

HF is acknowledged as the primary reason for mortality and incapacitation in the elderly population worldwide [26]. In truth, specific investigations propose that the outlook for HF is even graver when compared to the majority of malignancies [27]. HF profoundly diminishes the overall well-being of individuals and imposes a noteworthy economic burden on a global scale [28]. Despite the progress achieved in HF remedial agents, which have bolstered extended results [29–31], the clinical journey of HF continues to be unfavorable, emphasizing the need for novel diagnostic approaches to facilitate prompt detection and prevention.

In this research, an effective HF diagnostic model was developed and demonstrated a remarkable performance, achieving an AUV of 99.2% in the validated dataset. These findings are consistent with previously reported models [32]. Additionally, the hub genes identified in this model showed upregulation in our TAC mouse model. Notably, the HF diagnostic predictive model displayed superior diagnostic accuracy in patients with NICM compared to those with ICM. This discrepancy can be attributed to the distinct pathophysiological processes associated with myocardial fibrosis and conduction block in NICM and ICM, as well as the tissue cell specificity of the genes [8]. For example, oxidative stress levels differ significantly between patients with HF caused by ischemic and non-ischemic cardiomyopathy [33]. Although peripheral blood mononuclear cell samples are more readily obtainable, heart tissue samples provide a more precise representation of gene expression and can be acquired through biopsy or surgery in a clinical setting. Insight into the molecular mechanisms that underlie the development of HF helps to identify a range of abnormalities in cellular signaling pathways, which could potentially be targeted for diagnosis and treatment [34, 35].

Pathophysiological changes frequently occur prior to clinical symptoms, and the identification of distinct pathophysiological changes allows for early detection of the disease. Cardiac fibrosis is a prevalent pathological manifestation observed in both inherited and acquired heart diseases. Furthermore, it is recognized as the primary factor contributing to cardiac electrical conduction disorders and pump failure [36, 37]. The main objective of this study is to examine the essential genes linked to myocardial fibrosis and conduction block, as well as to explore the diagnostic model and potential mechanism of HF. Additionally, further investigation into the role of genes in the diagnostic model will significantly contribute to the diagnosis and treatment of HF.

### Some diagnostic model genes related to the mechanism of ECM formation

Our study aligns with previous investigations, as the DEGs in our research were predominantly implicated in biological processes and pathways linked to the ECM [38]. In the context of the heart, reactive oxygen species (ROS) triggers signaling pathways that are involved in cardiomyocyte hypertrophy, interstitial fibrosis, systolic dysfunction, and inflammation. These pathways have an impact on the structure and function of cells, ultimately resulting in heart damage and remodeling [39]. A study has shown that oxidative stress is associated with apoptosis, and inhibiting oxidative stress can potentially prevent the progression of dilated cardiomyopathy [40]. The phosphoinositol 3 kinase (PI3K) /Akt signaling pathway plays a crucial role in the development of cardiac fibrosis. It regulates various processes such as cell survival, apoptosis, growth, myocardial contractility, and transcription of relevant genes [41].

ECM is a complex network composed of a variety of biomolecules and extracellular matrix, which plays an important role in maintaining cardiac structure and function. Metabolic imbalance and excessive deposition of ECM are important features of myocardial fibrosis. The composition and structure of ECM can influence cell adhesion and migration, while cell adhesion and migration can regulate the formation and degradation of ECM. In our research, eight genes (ITGA3, ITGA8, ITGAL, PDGFRB, HIF1A, CXCL12, FGR, EGR1) are involved in various cellular processes such as adhesion, migration, and chemotaxis. These genes are closely linked to the formation of the ECM, specifically the pathways influenced by integrins. Four of these genes, FGR, ITGA3, ITGA8, and ITGAL, are essential components of integrin-mediated signaling. Integrins, which are receptor proteins on the cell membrane, play a critical role in facilitating communication between cardiac fibroblasts, cardiomyocytes, and the extracellular matrix (ECM) by responding to mechanical stress signals [42]. The stimulation of integrin signaling caused by pressure overload in the heart can result in the activation of myofibroblasts and excessive accumulation of collagen [43]. Prior studies have demonstrated that ITGA8 plays a role in promoting renal fibrosis by influencing fibroblast activation and immune cell infiltration [44]. Additionally, it has been shown that ITGA8 contributes to heart valve damage and repair through its impact on the interaction between myoblasts and the ECM [45]. Moreover, PDGFR-β has been observed to be activated via an integrin β1-dependent mechanism, promoting fibroblast proliferation and the synthesis of the ECM in a model of cardiac stress overload [46]. In numerous cardiac pathological states, the expansion of the ECM network or modifications in the composition of matrix proteins can result in heart disease. This may transpire either by directly impairing the heart’s normal structure and function or by transmitting inappropriate signals to cells [47]. The ECM governs diverse aspects of cell behavior within the cardiac microenvironment, encompassing attachment, movement, viability, growth, specialization, and maturation [48, 49]. cell adhesion, migration and cytokine chemotaxis plays an important role in ECM information.

HIF1A is an important transcription factor that plays a key role in the adaptive response of cells to low-oxygen environments. It is found that abnormal expression of HIF1A in the sympathetic nervous system affects myocardial collagen deposition, ECM formation, and myocardial fibrosis in diabetic cardiomyopathy [50]. HIF1A has been shown to enhance the activation of cardiac fibroblasts by upregulating the expression of specific genes associated with ECM formation [51]. Additionally, CXCL12 plays a crucial role in the chemotaxis of cardiac fibroblasts, thereby influencing cardiac remodeling [52]. Furthermore, it has been observed that FGR inhibitors stimulate the release of fibrotic chemokines, which in turn affects the deposition of collagen in tissues [53].

In summary, the diagnostic model gene exhibits characteristics that regulate cell adhesion, migration, and cytokine chemotaxis, and it is closely linked to ECM formation.

### Other model genes involved to inflammation and immune responses

In this study, we identified eight signature genes involved in immune and inflammatory responses: IL-10, TLR4, IFIT2, IFITM2, OAS2, STAT3, JAK1, and JAK2. IL-10 is an anti-inflammatory cytokine that regulates extracellular matrix biosynthesis and is predominantly found in T lymphocytes and macrophages [54]. IL-10 has been shown to have an anti-fibrotic effect by inhibiting the migration of fibroblast progenitor cells from the bone marrow to the heart and preventing their conversion into myofibroblasts [55]. Toll-like receptors (TLRs) are a family of pattern recognition receptors that play a crucial role in the innate immune system and are implicated in cardiovascular diseases. Among the ten TLRs found in humans, TLR4 is highly expressed in the heart. Activation of TLR4 in rat models has been shown to increase the production of IL-6 and ICAM-1, decrease cardiomyocyte contractility, and worsen HF. Conversely, inhibition of TLR4 reduces the expression of inflammatory mediators and improves cardiac function [56, 57].Surprisingly, in the present study, it was found that IFIT2, IFITM2, and OAS2, which were previously reported to be mainly involved in intrinsic cellular immune processes, are closely associated with HF. Previous studies have also reported the involvement of IFIT2 and IFITM2 in cardiac diseases. IFIT2 has been identified as a potential biomarker of ischemic cardiomyopathy in human and rat heart tissue samples [58], and it has been found to be highly expressed in HF patients with pulmonary arterial hypertension [59]. Additionally, studies on rat cardiomyocyte development have suggested that IFITM2 may play a role in differentiation and cell proliferation during cardiac development [60]. These findings indicate that inflammation and immune responses are key factors in the development of HF. Further investigation into the impact of each type of infiltrating immune cell may provide valuable insights for the development of new therapeutic strategies for HF.

### Possible relationship between immune cell infiltration and heart failure

Previous research has demonstrated that immune cell infiltration plays a critical role in the development and progression of cardiac disease [61, 62]. It has also been shown that immune cell infiltration of the myocardium can impair cardiac function [63–65]. Through single-cell sequencing analysis, the interaction between inflammation and myocardial fibrosis has been revealed, highlighting the role of immune cell infiltration in promoting the mechanis3m of HF [61, 66, 67].

In our study, we observed a correlation between the diagnostic genes and the level of immune cell infiltration. Specifically, we found that TLR4 exhibited a negative correlation with CD8 + T cells, while TLR4 showed a positive correlation with M2 macrophages. This is consistent with the findings of Gu et al., who identified TLR4 as a crucial target for regulating cardiac dysfunction after myocardial infarction, with its action being associated with macrophage activation [68]. Through the regulation of TLR4, Losartan effectively modulates macrophage polarization, resulting in a decrease in oxidative stress and cardiomyocyte apoptosis [69]. Consequently, sepsis-induced cardiomyopathy is alleviated. These findings underscore the substantial role of TLR4 in the process of macrophage polarization. Additionally, ITGAL has been found to be involved in various immune phenomena that contribute to the cytotoxicity of natural killer cells [70–73]. Previous studies [74, 75] have identified the association between OAS2 and T lymphocyte activation in psoriasis and systemic lupus erythematosus, indicating its role as an active phase marker of these diseases. However, limited attention has been given to the investigation of OAS2 in the context of heart failure. This current study reveals that OAS2 is significantly expressed in the myocardium of heart failure patients, presenting a fresh insight into the mechanism underlying HF. Collectively, these findings indicate that the diagnostic model genes can partially respond to the pathogenesis of HF, which laterally reflects the accuracy of the diagnostic model.

### Study limitations

While this diagnostic model demonstrated exceptional performance when applied to cardiac tissue samples from patients with NICM, its performance was mediocre when applied to peripheral blood mononuclear cells from patients with ICM. The diagnostic performance of this model in patients with peripheral blood mononuclear cells derived from NICM and in patients with cardiac tissue samples from ICM remains uncertain due to the lack of appropriate datasets. The limited availability of heart tissue samples may restrict the practical application of this model. It is important to note that our model was constructed and validated using expression profile data, but it has not been validated in prospective cohort studies. Therefore, further investigation is needed to determine its accuracy.

## Conclusions

In this study, we have identified 16 hub genes that are associated with myocardial fibrosis and conduction block phenotypes. Based on these findings, we have developed a diagnostic model for HF. These results have important implications for the intensive management of individuals who have underlying genetic variants linked to heart failure, especially in the context of advancing cell-targeted therapies for myocardial fibrosis.

### Electronic supplementary material

Below is the link to the electronic supplementary material.

Supplementary Material 1

## Data Availability

All data generated or analyzed during this study are included in this published article and its supplementary information files.All R code, and input/output data for this study are available in the figshare: 10.6084/m9.figshare.24586290.

## Abbreviations

HF: heart failure
DEGs: differentially expressed genes
ECM: extracellular matrix
ICM: ischemic cardiomyopathy
NICM: non-ischemic cardiomyopathy NICM
TAC: transverse-aortic constriction
LASSO: least absolute shrinkage and selection operator
SVM: support vector machines
GEO: Gene Expression Omnibus
GO: Gene Ontology
KEGG: Kyoto Encyclopedia of Genes and Genomes
GSEA: Gene set enrichment analysis
MF: molecular function
BP: biological process
CC: cellular component
ROC: receiver operating curves
AUC: area under the curve
NHF: non-HF
PPI: protein interaction network
