# Supplementary material for: Identification of diagnostic model in heart failure with myocardial fibrosis and conduction block by integrated gene co-expression network analysis
Source: BMC Med Genomics. 2024 Feb 14;17:52. doi: 10.1186/s12920-024-01814-w (PMC10868111; doi:10.1186/s12920-024-01814-w)
Supplement: Supplementary file 1 — Supplementary Material 1 [file 12920_2024_1814_MOESM1_ESM.doc]

Supplementary material


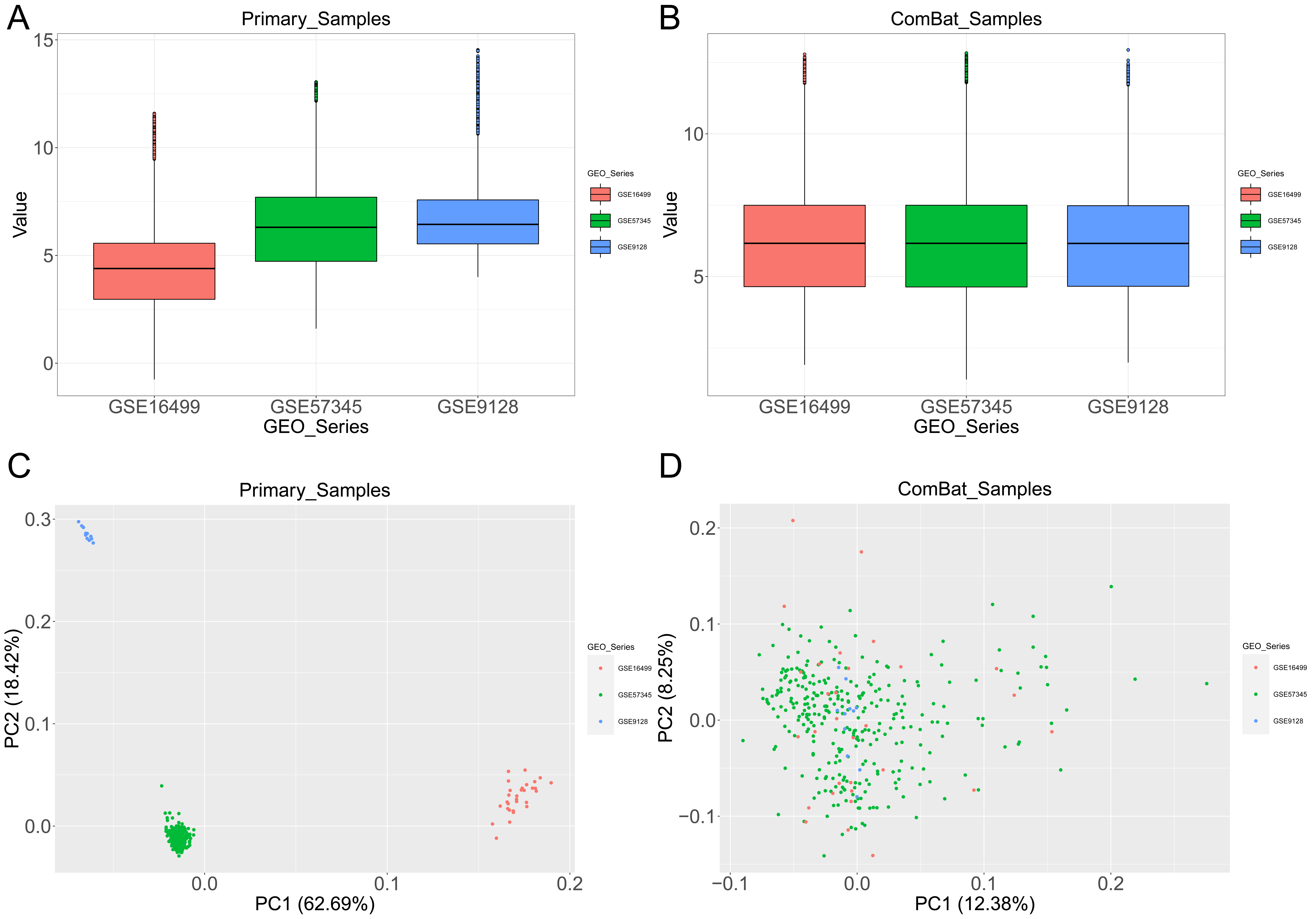


**Figure S1 Data preprocessing** (A) Box plot of the three merged data sets before correction. (B) Box plot of the three merged data sets after correction. (C) PCA analysis of the three data sets before batch correction with ComBat.(D) PCA analysis of the three data sets after batch correction with ComBat.
